# Innovative Machine Learning Approaches for Predicting the Asphalt Content During Marshall Design of Asphalt Mixtures

**DOI:** 10.3390/ma18071474

**Published:** 2025-03-26

**Authors:** Mutahar Al-Ammari, Ruikun Dong, Mohammed Nasser, Abdullah Al-Maswari

**Affiliations:** 1 School of Civil Engineering, Chongqing University, Chongqing 400045, China; l2400098@stu.cqu.edu.cn; 2 Department of Civil Engineering, Faculty of Engineering, Sana’a University, Sana’a 9671, Yemen; 3 Faculty of Engineering and Information Technology, Taiz University, Taiz 6803, Yemen

**Keywords:** machine learning, asphalt mixture (AC14), Marshall mix design

## Abstract

A flexible pavement with a proper Marshall mix design is essential for ensuring driving longevity, safety, and comfort. The increasing labor demands, costs, and time consumption for evaluating the Marshall mix design properties are due to extensive sample preparation, testing procedures, and material requirements. Consequently, this study aims to compare the conventional method of calculating the optimum asphalt content in Marshall mix design with machine learning approaches. This study focused on identifying the optimal asphalt content through the use of advanced machine learning methods, aiming to improve the accuracy of predicting the performance of asphalt mixtures. Therefore, this research investigates the application of various machine learning-based regression techniques to predict the properties of asphalt mixtures, focusing on evaluating their effectiveness in modeling this complex relationship. The main properties of interest include the Marshall stability, flow, VMA, VFA, and unit weight, all of which adhere to the Marshall mix design. A substantial database comprising 60 datasets was curated to aid in the development of these predictive models. Two stages were carried out in this research. The first stage was focused on determining the ideal asphalt content through conventional techniques, while the second stage involved comparing various algorithms to improve the prediction capabilities for asphalt pavement performance. At the end of the study, the comparisons of the various algorithms for the asphalt mixture parameters revealed that the neural network model outperformed all the others, achieving the highest accuracy based on R^2^ and MSE values. This highlights the neural network’s effectiveness in capturing the complexities of asphalt mixtures and its superior predictive capabilities compared to conventional methods, emphasizing its advantages in enhancing accuracy and reliability in asphalt mixture analysis.

## 1. Introduction

Machine learning is increasingly being used in asphalt mixture analysis to improve design, performance prediction, and quality control. Unlike traditional methods that are often slow and labor-intensive, machine learning provides a data-driven approach that boosts efficiency and accuracy. By analyzing historical data, machine learning models can identify patterns to predict key factors like rutting resistance and fatigue life, helping engineers optimize mixtures more effectively. Advances in data collection could enable real-time monitoring, transforming the field. By combining engineering expertise with machine learning, this study optimizes asphalt mix design, predicting parameters like the dry density, VMA, and VCA using models such as Random Forest (RF) and AdaBoost. RF excelled in predicting the dry density and VMA, while AdaBoost performed best for the VCA. The feature analysis highlighted the significant impact of aggregate gradation and compaction on predictions. This approach reduces testing time and costs, providing an efficient, data-driven solution to enhance asphalt mixtures and support sustainable infrastructure [1].

The integration of deep neural networks (DNNs) into the Mechanistic-Empirical Pavement Design Guide (MEPDG) has significantly enhanced asphalt pavement design by improving the accuracy of predictions for critical performance indicators, including rutting, fatigue cracking, and dynamic modulus [2]. Advanced models, including DNNs and artificial neural networks (ANNs), outperform traditional methods by addressing limitations such as neglecting key factors like air voids and reducing laboratory testing requirements [3]. These innovations optimize pavement design processes and contribute to more durable, cost-effective road infrastructure [4].

Recent developments in predicting the dynamic modulus of hot mix asphalt through deep learning techniques indicate that neural networks, especially neural network-viscosity based (NN-V) techniques, significantly surpass traditional models such as the Witczak model, particularly at higher values. This illustrates the effectiveness of deep learning in improving dynamic modulus estimation beyond conventional empirical approaches [5]. Furthermore, a study utilizing the M5P model tree algorithm creates predictive models by examining various influencing factors, which enhances performance for skewed data associated with asphalt composition [6]. Overall, bagged trees have also demonstrated superior capabilities compared to traditional regression and artificial neural networks in this field [7].

The existing literature has consistently demonstrated the effectiveness of machine learning in optimizing asphalt concrete and mixture predictions. Key findings indicate that methods such as Support Vector Regression and ensemble techniques effectively estimate the rut depth and indirect tensile strength, with extra trees performing best [8]. A Bayesian optimization process achieved a high prediction accuracy (R^2^ = 0.9909) for the stiffness of high-modulus asphalt [9]. Stochastic approaches using convolutional neural networks (CNNs) enhanced predictions for the dynamic modulus and phase angle, while the Catboost model excelled in dynamic modulus prediction (R^2^ = 0.998), outperforming eXtreme Gradient Boosting (XGBoost) and other methods [10]. Artificial neural networks (ANNs) also showed high accuracy (R^2^ = 0.989) in forecasting the dynamic modulus [11]. Additionally, techniques like Support Vector Machines effectively predicted the binder content in glass fiber-reinforced asphalt, and models such as augmented full quadratic and self-validated ensemble modeling reliably predicted the low-temperature fracture energy [12,13]. Gene expression programming (GEP) and hybrid models provided practical solutions [3]. Overall, these findings highlight the significant potential of machine learning to enhance asphalt mixture design and improve the pavement performance.

Earlier studies have laid the foundation for understanding asphalt mixtures through the application of machine learning (ML) techniques. Studies on asphalt binders modified with nanosilica (NS) and waste denim fiber (WDF) demonstrated significant improvements in rutting resistance, with optimal compositions identified at 4% NS and 5.1% WDF; ML models like Extreme Gradient Boosting Regression (XGBR) and Decision Tree Regression (DTR) achieved R^2^ values above 0.99, outperforming traditional response surface methodology (RSM) [14]. Additionally, advanced ML algorithms, including Support Vector Machine (SVM) and Random Forest (RF), were utilized to predict the optimal binder content in carbon fiber-reinforced asphalt, yielding high correlation coefficients and emphasizing the importance of parameters such as fiber length [15]. Furthermore, ML techniques were applied to streamline the prediction of effective and absorbed asphalt content, reducing the design time by 4–6 days [16]. Ensemble methods like Random Forest Regression (RFR) and XGBR were also employed to estimate the Marshall mix design parameters and resilient modulus for stabilized base materials, highlighting their potential to optimize material performance in pavement engineering [17].

Several studies demonstrate the effectiveness of machine learning in optimizing infrastructure materials and predicting pavement performance. These studies investigated improving the fracture toughness of asphalt rubber mixtures modified with wax and nano calcium carbonate, using multivariate regression and neural networks to optimize the additive content [18]. Machine learning was also applied to analyze the mechanical properties of fiber-reinforced polymer composites, identifying research trends and optimizing the material composition [19]. A machine learning model accurately correlated the moisture content and electrical parameters for evaluating the curing of emulsified asphalt chip seal, offering a potential method for rapid, non-destructive testing [20]. Furthermore, machine learning successfully predicted the asphalt mixture fracture load and pavement rutting depth, outperforming traditional methods [21,22]. Finally, a Bayesian-Ensemble Boosted Tree model effectively predicted flexible pavement fatigue cracking, highlighting the significance of traffic parameters. While data limitations and model interpretability remain challenges, these studies showcase the considerable potential of machine learning to enhance infrastructure design, assessment, and management [23].

Recent research has shown that machine learning models are effective in predicting pavement performance. An ensemble model achieved the best accuracy for forecasting the Pavement Condition Index (PCI) of asphalt overlays using Louisiana DOTD data, though the accuracy decreased over extended time frames [24]. Simultaneously, Gaussian Process Regression (GPR) and Linear Weighted Prediction (LWP) outperformed other models for predicting the International Roughness Index (IRI) in a significant Long-Term Pavement Performance (LTPP) dataset, with the initial IRI, pavement age, and subgrade plasticity as key factors [25]. Additionally, a novel gene expression programming (GEP) model excelled in predicting the rutting depth compared to ANN by effectively utilizing Hamburg wheel tracking test data. These findings highlight the potential of advanced machine learning techniques to enhance pavement management and predictive accuracy [26].

This research emphasizes the use of machine learning to optimize asphalt pavement performance. One study improves the asphalt mix design by predicting alligator and longitudinal cracking through a principal component analysis and ANN, outperforming traditional methods. Support Vector Regression (SVR) emerged as the most accurate method for predicting the air void content in asphalt layers, while deep residual neural networks (DRNNs) demonstrated a superior accuracy for predicting the dynamic modulus [27,28]. Additionally, machine learning models like AdaBoost and Random Forest successfully predicted Marshall characteristics, with AdaBoost showing the highest accuracy [29]. Research on modified asphalt binders with phase change materials (PCMs) indicated enhanced properties, and gene expression programming (GEP) assessed the impact of reclaimed asphalt pavement (RAP) and other materials on the rutting depth, confirming the significance of the RAP content [30,31]. These studies collectively showcase the potential of machine learning to enhance the design, durability, and sustainability of asphalt pavements.

While machine learning models have proven effective in predicting asphalt pavement performance, there remains a lack of research specifically targeting Marshall mixture design parameters. It is essential to explore various machine learning techniques to accurately predict asphalt properties, considering factors like aggregate types, gradation, and asphalt characteristics. This study focused on developing a model to predict the optimal asphalt content by examining different aggregate types and gradations. It utilized multiple machine learning methods, including linear regression, Bayesian Ridge Regression, Support Vector Regression (SVR), Decision Tree Regression, Random Forest Regression, Gradient Boosting Regression, K-Neighbors Regression, and Neural Network Regression, using 60/70 penetration grade asphalt. The findings aim to assist pavement engineers in making better-informed decisions about bitumen content when working with diverse aggregate types and gradations.

## 2. Methodology

**Figure 1:**
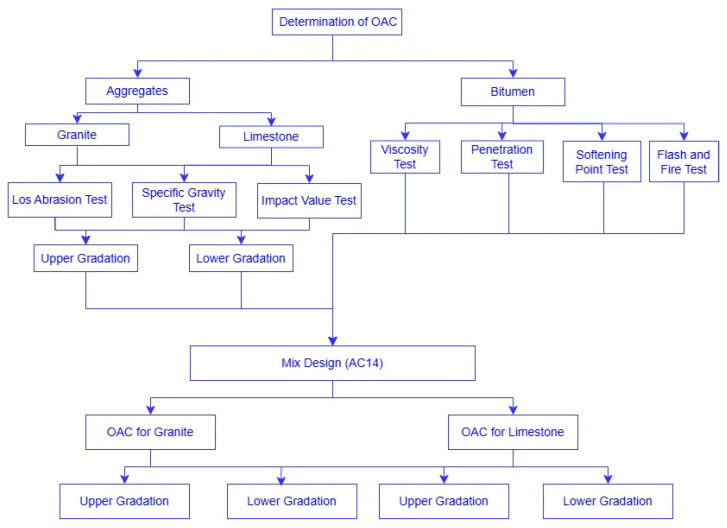
The flow chart for determination of OAC.

This investigation employed machine learning algorithms to comparatively assess asphalt mixtures utilizing granite and limestone aggregates, determining optimal asphalt content for each aggregate type across upper and lower bound gradations via Marshall mix design. The results of this optimal asphalt content determination are shown in Figure 1.

### 2.1. Sample Preparation

This study focused on preparing asphalt mixtures using granite and limestone aggregates, 60–70 penetration grade asphalt, and fillers adhering to JKR 2008 standards. Before blending, the aggregates underwent rigorous testing, including Los Angeles Abrasion, impact value, and specific gravity tests, followed by sieving to meet the ACW14 gradation specified by JKR 2008. The asphalt was tested according to ASTM standards for penetration, viscosity, softening point, and flash/fire point. Using the traditional Marshall mix design method, 60 samples were prepared with 75 compaction blows to determine the optimal asphalt content, ensuring an effective mixture composition.

### 2.2. Machine Learning Models and Analysis

The study investigated the use of machine learning algorithms, especially neural networks, for modeling and optimizing bitumen content to improve asphalt characteristics. Researchers analyzed a dataset of 60 records, which were categorized into four groups based on aggregate type (limestone and granite) further divided into lower and upper gradation. The goal was to identify the most effective machine learning model for each characteristic, while also comparing the performance of traditional methods against advanced predictive techniques.

### 2.3. Differential Evolution Algorithm

The Differential Evolution (DE) Algorithm is an optimization method designed to find the best solution to a problem by iteratively improving a population of candidate solutions over several generations. It works by evolving a group of individuals (solutions) through processes like mutation, crossover, and selection. The goal is to identify the values of input variables that either maximize or minimize a given objective function while adhering to specific constraints.

In this study, the DE algorithm is employed to ascertain the ideal amount of bitumen that results in the best asphalt mixture characteristics. Specifically, the algorithm seeks to maximize the bulk specific gravity (Gmb) while adhering to specific constraints derived from models that predict other asphalt mixtures properties. The DE algorithm will iteratively modify the bitumen content and additional input parameters within defined ranges, using the models to assess the resulting asphalt mixture characteristics, ultimately providing the bitumen amount that maximizes the bulk specific gravity while meeting the established constraints.

## 3. Key Steps in the Differential Evolution Algorithm

1. Initialization: A population of candidate solutions is randomly generated within specified bounds (minimum and maximum values for the asphalt amount and other properties).
2. Mutation: For each candidate solution, a new candidate is created by applying mutation, which typically involves adding weighted differences between two randomly selected solutions to a third solution.
3. Crossover: The newly mutated candidate is combined with the original candidate to form a new solution. The crossover operation defines the proportion of the original and mutated solutions retained in the new candidate.
4. Selection: The new candidate is evaluated against the original solution, and the superior solution—based on the objective function, such as maximizing bulk specific gravity—is selected for the next generation.
5. Iteration: These steps are repeated over multiple generations until a convergence criterion is achieved.

**Figure 2:**
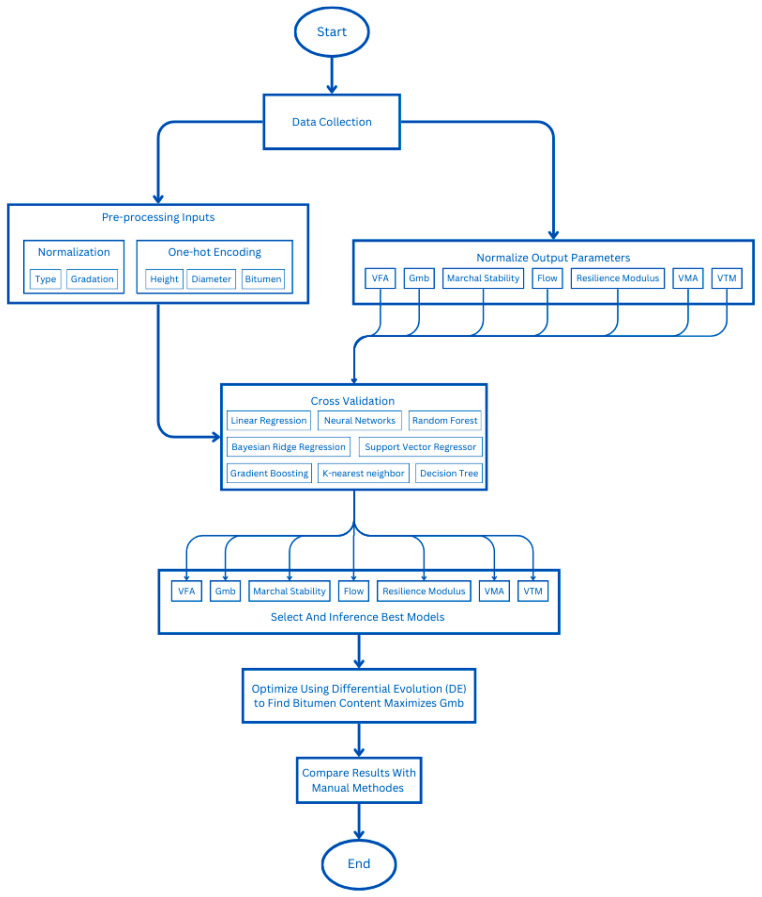
The flow chart for machine learning models.

The objective function is expressed as a machine learning model that has been trained using experimental data outputs to optimize bulk specific gravity. The outcomes of the machine learning models are illustrated in Figure 2.

### 3.1. Data Preprocessing

A total of six distinct characteristics (flow, Marshall stability, voids filled with asphalt (VFA), voids in total mix (VTM), resilient modulus, and bulk specific gravity) were systematically modeled using an array of machine learning algorithms to determine the most proficient predictive model for each characteristic. The modeling process employed the gradation, bitumen content, height, and diameter as input variables, enabling a thorough analysis of the interrelationships and factors that impact these critical performance indicators in asphalt mixtures.

One-hot encoding was implemented for categorical variables, including type and gradation, while normalization techniques were applied to numerical data fields, such as the bitumen content, height, and diameter. Furthermore, normalization was also utilized for each regression output. The normalization process is mathematically represented by the following equation:

$$X_{normalized}=\frac{X-\mu }{\sigma }$$

where

*X* is the original value, *μ* is the mean of the dataset, and *σ* is the standard deviation of the dataset.

### 3.2. Linear Regression

Linear regression operates under the premise of a linear relationship between the input variables and the target output, determining a best-fit line that minimizes the sum of squared errors. The predicted output ($\hat{y}$) is calculated as a weighted sum of the input features (X), supplemented by a bias term (β). While this model is well suited for data exhibiting a linear trend, it may encounter challenges when dealing with non-linear relationships.

$$\hat{y}=\beta_{0}+\beta_{1}X_{1}+\beta_{2}X_{2}+\cdots +\beta_{n}X_{n}$$

### 3.3. Bayesian Ridge Regression

Bayesian Ridge Regression enhances linear regression by employing Bayesian inference to estimate a distribution for the model’s coefficients, which introduces regularization to mitigate the risk of overfitting. This model strikes a balance between fitting the data and maintaining complexity, making it particularly effective for datasets with high variance or numerous features. The formulation closely resembles that of linear regression, with the inclusion of prior distributions on the weights.

### 3.4. Support Vector Regressor (SVR)

Support Vector Regressor (SVR) conducts regression by identifying a hyperplane that optimally fits the data within a defined margin of tolerance. The goal of SVR is to encompass as many data points as possible within this acceptable range, with particular emphasis on those that are closest to the margin, known as support vectors. This method is particularly effective at modeling non-linear relationships when used in conjunction with kernel functions.

$$f\left(X\right)=\sum_{i=1}^{n}\alpha_{i}K(X,X_{i})+b$$

where K is the kernel function, α are coefficients, and b is a bias term.

### 3.5. Decision Tree Regressor

A Decision Tree Regressor constructs a tree-like structure by dividing the data into subsets according to feature values, with each division aimed at minimizing the prediction error. The tree culminates in leaf nodes, which represent the model’s predictions. While this approach is straightforward and interpretable, it is prone to overfitting, particularly when the trees are overly deep.

$$f\left(X\right)=\sum_{i=1}^{L}\frac{1}{N_{i}}\sum_{j\in N_{i}}y_{j}$$

where L is the number of leaves, Ni is the number of observations in each leaf, and yj represents observed values in each leaf.

### 3.6. Random Forest Regressor

The Random Forest Regressor creates an ensemble of Decision Trees, with each tree trained on a random combination of the data and features. By averaging the predictions from these trees, the model reduces variance and enhances accuracy. This methodology helps to prevent overfitting, rendering the model more resilient to outliers and noise.

$$\hat{y}=\frac{1}{M}\sum_{m=1}^{M}f_{m}(X)$$

where M is the number of trees and $f_{m}$ represents the prediction from each individual tree.

### 3.7. Gradient Boosting Regressor

The Gradient Boosting Regressor is an ensemble technique that incrementally constructs a series of trees, with each new tree addressing the errors made by its predecessor. Beginning with an initial model, subsequent trees focus on minimizing residual errors, resulting in a highly accurate model that is well suited for complex datasets.

$${\hat{y}}_{i}=y_{i}^{\left(0\right)}+\sum_{m=1}^{M}\nu f_{m}(X_{i})$$

where $y_{i}^{\left(0\right)}$ is the initial prediction, M is the number of trees, $f_{m}$ is each subsequent model, and ν is the learning rate.

### 3.8. K-Neighbors Regressor

The K-Neighbors Regressor predicts the output for a new data point by calculating the average of the outputs from its (k) nearest neighbors within the training set. This non-parametric method is effective for small datasets; however, it may encounter difficulties when dealing with high-dimensional data.

$$\hat{y}=\frac{1}{k}\sum_{i=1}^{k}y_{i}$$

where k is the number of nearest neighbors and $y_{i}$ are the outputs of these neighbors.

### 3.9. Neural Network Regressor

Neural networks (NNs) are a category of machine learning models inspired by the architecture of the human brain, comprising layers of interconnected neurons. These layers function to transform input data into output predictions, with each layer progressively learning to identify more complex features. A standard neural network includes an input layer, one or more hidden layers, and an output layer. The architecture of the network, which includes the number of layers and neurons, can be modified to address the complexity of the data. For regression tasks, the output layer typically features a single neuron that generates continuous values, establishing neural networks as a potent tool for predicting outcomes with intricate, non-linear relationships.

A Neural Network Regressor is a deep learning model structured with layers of interconnected neurons, which adaptively learn complex patterns by modifying weights to minimize prediction errors. The model utilizes a technique called backpropagation, wherein the error is transmitted backward through the layers to refine the weights, ultimately enhancing the precision of its predictions.

$$output=f(\sum_{i=1}^{n}\omega_{i}x_{i}+b)$$

where f is an activation function, Wi are weights, xi are inputs, and b is a bias term.

Training a neural network involves adjusting its weights and biases using an optimization process called backpropagation. During training, the model compares its predictions to actual values, calculates the error using a loss function like Mean Squared Error (MSE), and propagates this error backward to update the weights. The goal is to minimize the loss function by fine-tuning the model’s parameters, enhancing prediction accuracy. Neural networks are highly effective at capturing complex patterns in data, making them ideal for regression tasks where simpler models, such as linear models, may fall short in handling intricate relationships.

## 4. Results and Discussion

### 4.1. First Phase: Marshall Parameter Testing

This method was employed to ascertain the optimum asphalt content (OAC) for each mixture type, as illustrated in Figure 1. The specimens were evaluated to determine the OAC using parameters such as density, 4% air voids, stability, and resilient modulus. The results, consistent with the UPM method, are detailed in Table 1.

The results showed that the granite mixtures, in both the upper and lower gradations, utilized a higher percentage of asphalt content compared to limestone mixtures within the same gradations. Additionally, the asphalt content was found to be higher in lower gradation mixtures than in upper gradation mixtures.

#### Analysis Tests for (OAC)

**Figure 3:**
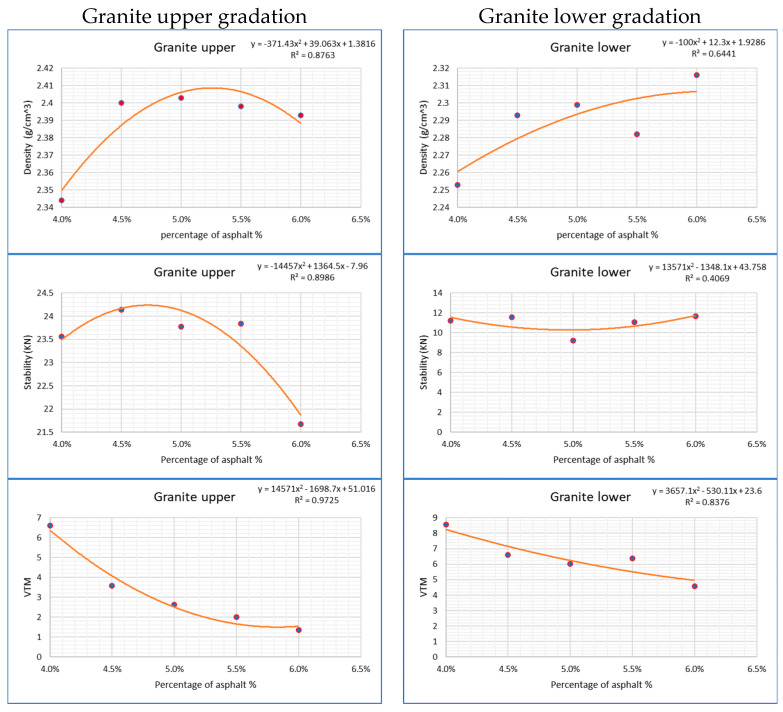

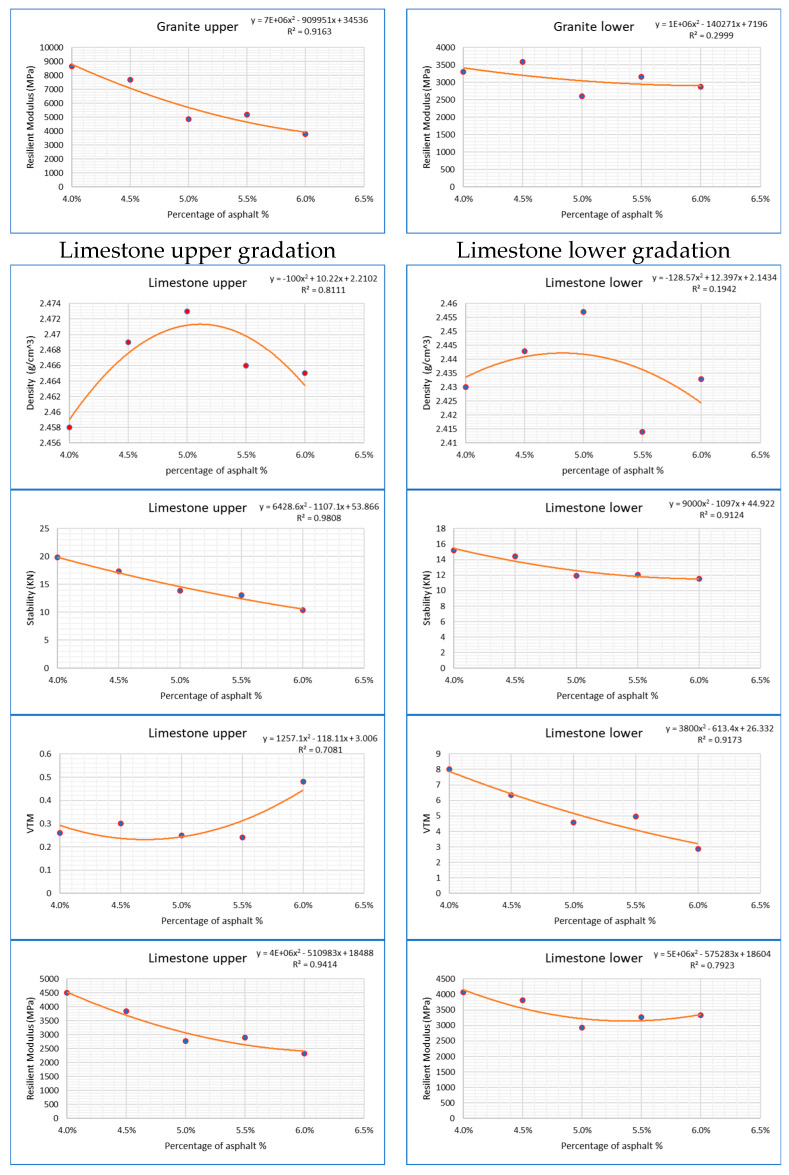
Laboratory analysis of Marshall mix parameters.

The asphalt mixture evaluated two types of aggregates in both upper and lower gradations. Laboratory tests were performed on samples from each mixture to assess the bulk specific gravity, stability, VTM, and resilient modulus values. The findings are presented in Figure 3.

The Marshall properties were compiled based on the optimum asphalt content and the results of the Marshall test analysis conducted in the first phase, using the Marshall procedure to validate adherence to the JKR/SPJ/2008 standards listed in Table 2.

According to the results in the table above, the stability and stiffness values conform to the ranges defined in JKR specifications. However, only the VTM values for the granite upper gradation mixture met the specified range.

### 4.2. Second Phase: Analysis of Machine Learning Models

The main aim of this approach was to carry out a comparative analysis of different algorithms used to predict the properties of asphalt mixtures. This included evaluating traditional techniques such as linear regression and Bayesian Ridge Regression, as well as advanced methods like Support Vector Regression (SVR), Decision Tree Regression, and Random Forest Regression. The assessment also incorporated Gradient Boosting Regression, K-Neighbors Regression, and Neural Network Regression. By investigating the effectiveness of these various algorithms, the goal was to identify the most accurate method for predicting asphalt mixture properties, which would ultimately improve pavement design and management.

#### 4.2.1. Cross-Validation

Cross-validation is a method employed to evaluate the performance of a machine learning model by dividing the dataset into several subsets, known as “folds”. In this study, a 10-fold cross-validation approach was utilized due to the limited size of the dataset. In this procedure, the dataset is randomly partitioned into 10 equal segments. The model is trained using 9 of these segments while testing is performed on the remaining segment, with this process repeated 10 times, each time designating a different segment as the test set. The outcomes from all 10 iterations are then averaged to provide a more robust assessment of the model’s performance.

Gauging the predictive performance of the models involved the use of two evaluation metrics: Mean Squared Error (MSE) and R-squared (R^2^).

The Mean Squared Error (MSE) assesses the average squared difference between the predicted and actual values, with a lower MSE indicating improved performance (Table 3). The formula for calculating MSE is as follows:

$$MSE=\frac{1}{n}\sum_{i=1}^{n}{\left(y_{i}-{\hat{y}}_{i}\right)}^{2}$$

where $y_{i}$ is the actual value, ${\tilde{y}}_{i}$ is the predicted value, and n is the number of data points.

The R-squared (R^2^) metric measures the degree to which the model explains the variability of the target variable. It ranges from 0 to 1, where a value closer to 1 suggests a superior fit (Table 4). The equation for R^2^ is as follows:

$$R^{2}=1-\frac{\sum_{i=1}^{n}{\left(y_{i}-{\hat{y}}_{i}\right)}^{2}}{\sum_{i=1}^{n}{\left(y_{i}-\bar{y}\right)}^{2}}$$

where $y_{i}$ is the actual value, ${\tilde{y}}_{i}$ is the predicted value, and $\bar{y}$ is the mean of the actual values.

The performance of the developed models was assessed through various statistical metrics, specifically the Mean Squared Error (MSE) and R-squared (R^2^). The R^2^ value ranges from 0 to 1, where higher values signify a more accurate model with predictions closely aligned with actual measurements. Conversely, a lower Mean Squared Error (MSE) reflects improved model performance by indicating fewer prediction errors. Among all the models evaluated, the neural network model demonstrated the best performance.

#### 4.2.2. Neural Network Regression (NNR)

**Figure 4:**
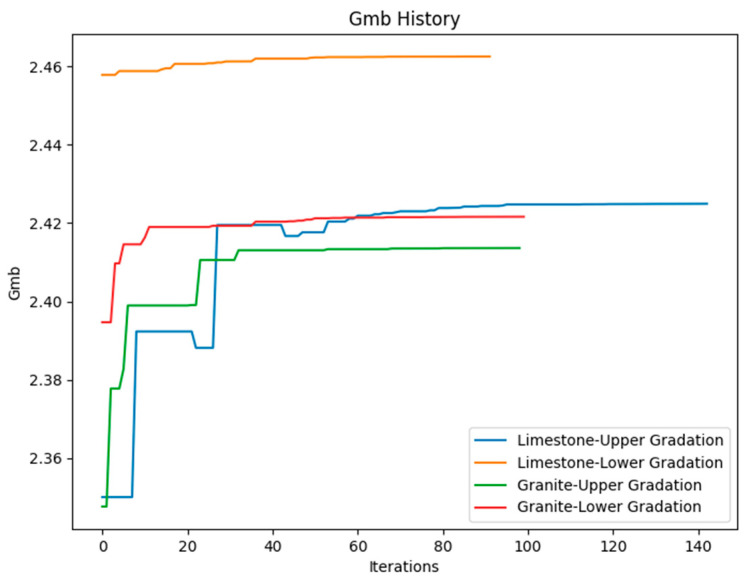
Optimization process using Differential Evolution Algorithm.

This study identified the neural network model as the most accurate prediction method for improving asphalt mixtures, while also optimizing these models using Differential Evolution techniques. The results illustrate the iterations and corresponding Gmb values throughout the optimization process, as shown in Figure 4.

The optimization process was carried out for the four types and gradations, as shown in Figure 4. The best bitumen quantities were determined for each of the four scenarios, and the results are displayed in Table 5.

**Figure 5:**
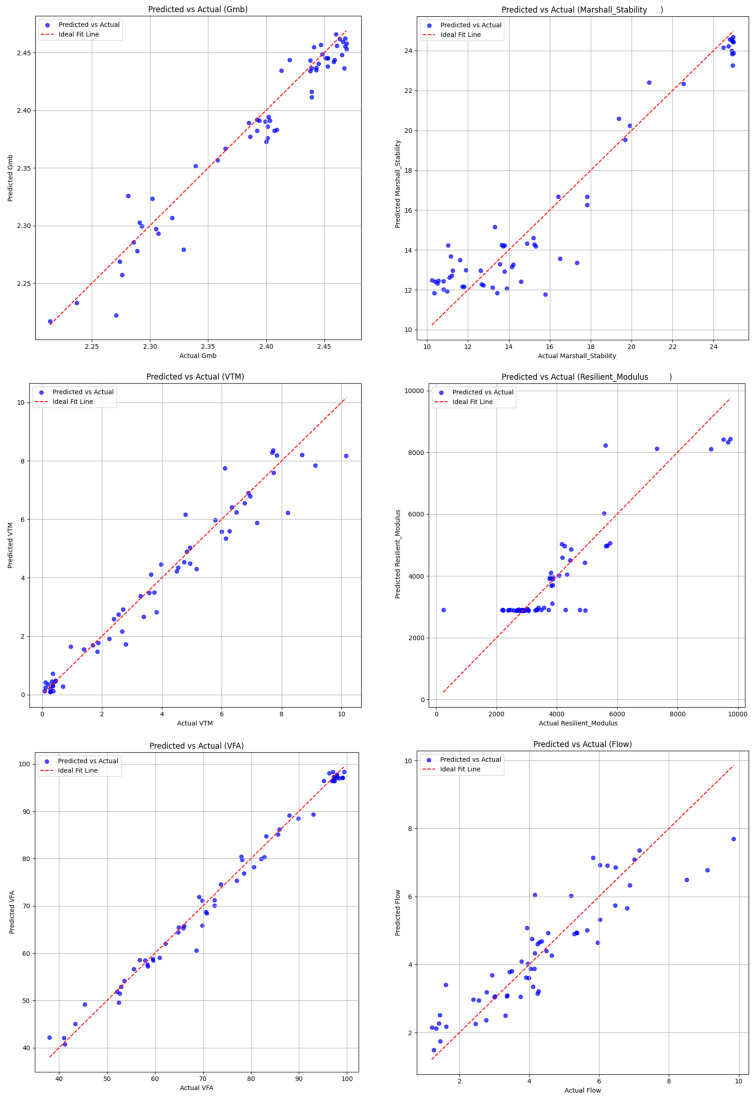
Actual and predicted Marshall mix design parameters from the NN model.

The performance of the neural network models in comparing the actual and predicted values of Marshall mix design properties is illustrated in Figure 5. 

#### 4.2.3. Summary of Results

**Figure 6:**
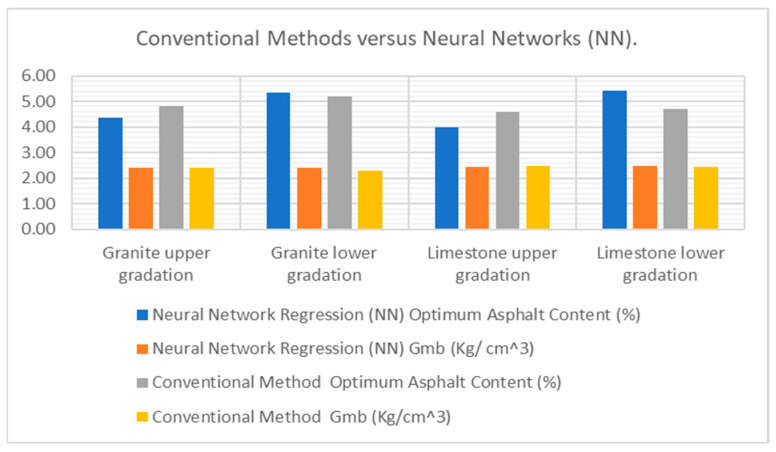
Comparison of performance metrics for OAC and Gmb using conventional methods versus neural networks (NNs).

The neural network model provided the most accurate predictions for both the actual and estimated Marshall mix design parameters. The results highlighted a comparison of the optimum asphalt content (OAC) and bulk specific gravity (Gmb) values generated by the neural network with those obtained from traditional methods, as presented in Figure 6.

## 5. Conclusions

This study investigated how different aggregate gradations affect asphalt mixture performance and evaluated predictive modeling approaches for key parameters. The analysis showed that granite-based mixtures outperformed their limestone counterparts, with the upper gradation granite demonstrating a superior strength (24.23 KN stability) and flexibility (6200 MPa resilient modulus). While the lower gradation granite required more binder, it proved less durable. The limestone mixtures showed a varied performance, with lower gradation exhibiting the weakest structural properties but a higher air void content, highlighting how material selection and particle distribution critically influence pavement quality.

In predictive modeling comparisons, neural networks emerged as the most accurate tool for forecasting asphalt characteristics like the optimal binder content and density. These AI-driven models achieved higher R^2^ values (indicating better fit) and lower prediction errors (MSE) than traditional methods. The visual data comparisons clearly demonstrated the neural networks’ ability to handle complex material interactions that often challenge conventional analysis techniques.

The research confirms neural networks’ potential to revolutionize asphalt design processes. By delivering precise predictions for both granite and limestone mixtures across gradation ranges, these models enable engineers to create more durable pavements while reducing costly trial-and-error testing. For example, improved accuracy in determining the optimal asphalt content could help prevent common issues like rutting or cracking, extending roadway lifespans and lowering maintenance budgets.

These findings address a critical need in infrastructure development, where material optimization directly impacts project costs and sustainability. The models allow engineers to fine-tune mixtures for specific climate conditions or traffic loads while minimizing expensive over-engineering. This precision becomes particularly valuable for megaprojects, where a 1–2% improvement in material efficiency could translate to six-figure savings without compromising quality.

While promising, the models’ reliability depends on comprehensive training data. Current limitations include the study’s focus on granite/limestone blends and controlled lab conditions. Future work should incorporate diverse materials (like recycled aggregates) and real-world environmental factors. Exploring hybrid models that combine neural networks with physics-based simulations could further enhance predictive capabilities, potentially unlocking new standards for smart pavement design.

By bridging materials science with machine learning, this work provides both immediate tools for industry practitioners and a roadmap for developing next-generation civil engineering solutions. The integration of AI into asphalt analysis not only improves current practices but also opens doors to data-driven innovations in infrastructure resilience.

## Figures and Tables

**Table 1:** Optimum asphalt content (OAC).

Type of Aggregate	OAC (%) for Density	OAC (%) for Stability	OAC (%) for VTM	OAC (%) for Resilient Modulus	Average Optimum Asphalt Content
Granite upper gradation	5.3	4.7	4.5	4.5	4.8
Granite lower gradation	6	5	6.8 *	4.5	5.2
Limestone upper gradation	5.1	4.3	NA	4.3	4.6
Limestone lower gradation	4.8	4.3	5.5	4	4.7

* Value not considered in calculation. NA: Not applicable. Red indicates the aggregate type in asphalt mixtures, while blue highlights the parameters for determining OAC.

**Table 2:** Summary of Marshall test results.

Type of Mixture	OAC (%)	Density (Kg/cm^3^)	Stability (KN)	VTM (%)	Resilient Modulus (MPa)
JKR/SPJ/2008 Specification	4–6	-	>8 KN	3–5	>2000
Granite upper gradation	4.8	2.401	24.23	3.05	6200
Granite lower gradation	5.2	2.298	12.49	5.92	3100
Limestone upper gradation	4.6	2.469	16.1	NA	3800
Limestone lower gradation	4.7	2.442	12.53	5.9	3845

NA: Not applicable. Red indicates the aggregate type in asphalt mixtures, while blue highlights the parameters for determining OAC.

**Table 3:** Gauging the predictive performance of the models using MSE.

Model	Flow MSE	G_mb_ MSE	Marshall Stability MSE	Resilient Modulus MSE	VFA MSE	VMA MSE	VTM MSE
Linear Regression	1.1282	0.0003	8.6917	2,022,443.2421	89.3324	0.3683	1.8881
Bayesian Ridge Regression	1.1212	0.0003	8.6839	1,998,274.0914	89.1263	0.3692	1.8676
Support Vector Regressor (SVR)	1.3895	0.0007	7.2507	1,327,800.8528	42.2544	0.7462	1.3124
Decision Tree Regressor	2.5272	0.0005	4.2419	1,098,981.8458	41.1321	0.6064	1.4373
Random Forest Regressor	1.6286	0.0003	2.9487	896,862.0808	30.2089	0.3532	1.1686
Gradient Boosting Regressor	1.7801	0.0004	4.0591	858,300.1841	25.6149	0.5399	1.2474
K-Neighbors Regressor	1.0595	0.0004	3.8122	1,404,392.5592	60.3673	0.6225	1.5999
Neural Network Regressor	0.9493	0.0003	2.9841	893,475.1084	15.4153	0.3753	0.8226

In asphalt mixtures, red denotes algorithms, and blue indicates OAC parameters for determining OAC.

**Table 4:** Gauging the predictive performance of the models using R-squared (R^2^).

Model	Flow	G_mb_	Marshall Stability	Resilient Modulus	VFA	VMA	VTM
	R^2^	R^2^	R^2^	R^2^	R^2^	R^2^	R^2^
Linear Regression	0.6952	0.9432	0.6848	0.4133	0.7293	0.8827	0.7636
Bayesian Ridge Regression	0.6971	0.9433	0.6851	0.4203	0.7299	0.8825	0.7662
Support Vector Regressor (SVR)	0.6246	0.8655	0.7371	0.6148	0.8719	0.7624	0.8357
Decision Tree Regressor	0.3173	0.8922	0.8462	0.6812	0.8753	0.8068	0.8201
Random Forest Regressor	0.56	0.9384	0.8931	0.7398	0.9085	0.8885	0.8537
Gradient Boosting Regressor	0.5191	0.9187	0.8528	0.751	0.9224	0.828	0.8438
K-Neighbors Regressor	0.7138	0.914	0.8618	0.5926	0.8171	0.8017	0.7997
Neural Network Regressor	0.7435	0.9408	0.8918	0.7408	0.9533	0.8805	0.897

In asphalt mixtures, red denotes algorithms, and blue indicates OAC parameters for determining OAC.

**Table 5:** Optimum asphalt content (OAC) using NNR.

Type of Aggregate	Optimum Asphalt Content (%)	Height (mm)	Diameter (mm)	Gmb
Granite upper gradation	4.35027885	62.7005655	101.0006294	2.4136278
Granite lower gradation	5.3300419	62.7009352	101.6999504	2.4216117
Limestone upper gradation	4.00002129	64.0433294	101.0000297	2.4249282
Limestone lower gradation	5.40446488	62.7000285	101.6998767	2.4624772

Red indicates the aggregate type in asphalt mixtures.

## Data Availability

The data presented in this study are available on request from the corresponding author due to ethical restrictions imposed to protect the privacy and confidentiality of human participants.
